# The Need for Online Information on the Economic Consequences of Cancer Diagnosis, Treatment, and Survivorship

**DOI:** 10.2196/jmir.7.3.e29

**Published:** 2005-07-01

**Authors:** Cathy J Bradley

**Affiliations:** ^1^Department of Health Administration and Massey Cancer CenterVirginia Commonwealth UniversityRichmond, VAUSA

**Keywords:** Internet, cancer, health care costs

## Abstract

The Internet is commonly used to provide treatment information to patients diagnosed with cancer. Notably missing from the existing websites is information on the cost of cancer care in terms of medical costs to the patient and work-related consequences. The purpose of this paper is to describe what is known about the economic cost of cancer and to describe how this information can be structured so that it is of more benefit to patients. This paper first provides an overview of the information available regarding medical expenses and productivity costs associated with cancer survivorship, particularly with respect to cancer and employment. Second, it draws attention to the sparse economic information available online to cancer survivors. Patients can find information on sources of financial assistance, but they cannot estimate from the available information the cost of their care or anticipate the impact that cancer and its treatment may have on their jobs. Finally, a strategy for filling the void in online economic cancer information is described. Substantial opportunity exists to provide economic information to cancer patients and their families. The Internet is a natural forum for gathering and disseminating economic information. A unique advantage of the Internet is its ability to put information immediately in the hands of cancer patients and their families—assisting them to become informed consumers and skilled negotiators.

## Introduction

The US National Cancer Institute (NCI) has made several calls for research with regard to the economic aspects of cancer diagnosis, treatment, and, ultimately, survivorship. A major impetus for such research is that the prevalence of early stage disease is rising and the number of long-term survivors now approaches 10 million as many cancers are becoming chronic conditions. Yet our understanding of how newly diagnosed cancer affects the economic viability of survivors and their families is remarkably incomplete. Economic information is largely absent from common Internet websites that offer information to cancer patients and their families—leaving patients in the untenable position of having to make treatment choices without fully understanding the costs and the impact on their ability to work.

Two important dimensions of economic data—medical and productivity costs—are relevant to patients, physicians, and society. This paper takes the patient's point of view. Direct medical costs are defined as the cost of medical care, including inpatient, outpatient, physician and other provider services, pharmaceuticals, and supportive care. From a patient's perspective, these costs are highly relevant since the costs associated with cancer care can be very expensive and perhaps prohibitive—even for patients who have generous health insurance benefits. As these costs rise, physicians and other health care providers may find themselves in the position of discussing with patients the trade-offs of treatment in terms of their relative costs and benefits. As aptly noted by Fryback and Craig, “Sooner or later a balance must be struck between the cost of interventions and their effectiveness” [1].

Productivity costs are defined as the time loss from work or the inability to fully function on the job when present. Documenting health-related economic losses is of great interest to patients and employers, who share the economic burden of illness. The probability of developing cancer is 1 in 12 for individuals aged 40 to 59 [2] and is likely to rise as screening is routinely recommended for younger individuals. Thus, the need for information that can assist this growing population of cancer survivors to minimize the economic consequences of their treatment decisions is vital.

The purpose of this paper is to describe what is known about the economic cost of cancer and to describe how this information can be structured so that it is of more benefit to patients. This paper first provides an overview of the information available regarding medical expenses and productivity costs associated with cancer survivorship, particularly with respect to cancer and employment. Second, it draws attention to the sparse economic information available online to cancer survivors. Finally, a strategy for filling the void in online economic cancer information is described.

## Economic Cost of Cancer

### Treatment Costs

Nationally, the direct cost of cancer care was approximately US $60.9 billion in 2002 [3]. However, at the individual patient level, we know very little about the cost of cancer care [1]. In the absence of clinical consensus in favor of one treatment (as is becoming the case with routine use of combination therapies), cost factors become more important in the treatment decision. Among the many studies of cancer cost found in the literature [3-16], few studies describe cost in terms of burden to patients. More commonly, studies approach cancer care cost estimation by segmenting the course of disease into phases (eg, first 6 months after diagnosis, the last 6 or 12 months of life, and the time between the first and last intervals) [8-11]. Other studies address isolated points in the range of cancer sites and treatments [4,5,12].

These studies provide useful information to health care providers, payers, and perhaps policy makers, but they are less beneficial to patients who need to consider cost in their treatment decisions. In addition, these studies fall far short of describing the range of treatment options available for different types of cancer. Fryback and Craig argue that, in many cancer interventions, the patient can be considered a provider of care along with the oncologist [1]. Patients and their families must find time and financial resources to complete lengthy treatment protocols that often involve toxic side effects and short-term disability.

Because not all costs apply to all patients due to variations in health insurance benefits and other financial arrangements, the current methods used for collecting and estimating economic cost are not useful to patients. Thus, alternative methods for estimating the economic costs for patients and families are required. In the scientific literature, some studies have counted resources used to treat patients for cancer [13-15]. A cost can be applied to these “counts” of resources depending on the patient and treatment scenario offered. Rizzo et al [16] used a validated and reliable questionnaire for purposes of collecting patient-level costs for patients undergoing bone marrow transplants. By and large, such information regarding patient costs is not widely available and little is known about how to collect, organize, and analyze patient-level costs [17].

The economic burden on patients and their families for cancer treatment may include the immediate cost of treatment, out-of-pocket expenses (eg, supportive care medication, co-payments, child care), and future costs required for cancer surveillance, follow-up care, and treatment of persistent symptoms (eg, pain, fatigue) [18]. Out-of-pocket expenses will be incurred by all patients, and these costs can vary widely depending on where the patient lives and shops. For example, prescription drug costs vary from local pharmacies, to discount pharmacies, to Internet pharmacies. Other out-of-pocket costs include transportation, child care, and home care services—all of which can add to a significant amount of money. More significantly, out-of-pocket expenses can also include the cost of participation in a clinical trial.

The availability of economic information can greatly affect health outcomes. For example, a woman choosing between mastectomy and breast-conserving surgery needs to know if she can afford chemotherapy, radiation, and tamoxifen following lumpectomy. She and her family must consider the resources (eg, transportation, time away from work, child care) required to complete radiation and chemotherapy. If she cannot complete the care regimen following lumpectomy because of financial concerns, mastectomy may be a more optimal choice for her long-term survival. On the other hand, if the woman chooses a lumpectomy because of rapid recovery time and lower immediate costs, but later becomes non-adherent to chemotherapy and radiation therapy, she will jeopardize her long-term health. Unfortunately, less than optimal treatment choices are likely to be made by patients who have the fewest resources to rely upon.

Incorporating an economic dimension into cancer care raises deeply rooted ethical concerns and contradicts a notion of cure at any cost. However, ignoring the financial burden of cancer care may jeopardize patient outcomes if patients choose a course of treatment but alter the dose (as they can with oral medications) or prematurely cease treatment. Patients and their families need to consider treatment choices in light of economic costs.

### Work Loss

Turning the discussion to work loss, the literature is unequivocal about work loss attributable to cancer. In addition, as more and more working-age individuals are screened for cancer, employed, as opposed to retired individuals, will be treated for cancer. For example, the US Preventive Services Task Force found evidence that annual prostate cancer screening can detect early-stage prostate cancer in men age 50 and over [19]. For African American and asymptomatic men with a family history of prostate cancer, screening is recommended to start at age 40 [20].

Some studies [21-26] have focused on survivors' subjective impressions of the impact of cancer on their lives. These studies suggest several negative factors that can reduce employment, including physical disability (eg, limitations in upper body strength [22]), memory loss [27], lack of control over schedules, need for transportation, type of work performed [28,29], and, in some cases, discrimination on the part of employers [30]. Chirikos et al [31], in their study of 5-year breast cancer survivors, reported that 41% required special accommodations to perform their jobs. These survivors were nearly three times more likely to be impaired relative to their non-cancer peers. The literature on the impact of cancer on work does not extend to cancer's impact on productivity for employed patients who continue to work while undergoing treatment. While it would not be surprising that treatments such as chemotherapy lower productivity, the absence of estimates regarding the amount and duration of productivity losses is somewhat remarkable.

Research using data from the Health and Retirement Study examined labor market participation, wages, and earnings of breast cancer survivors relative to a nationally representative non-cancer control group [32,33]. These women were statistically significantly less likely to work (by approximately 9 percentage points) relative to women who never had cancer. A more recent study examined post-treatment changes in labor supply among women working prior to a breast cancer diagnosis and among men working prior to a prostate cancer diagnosis relative to a control group of initially working women and men. Women with breast cancer were about 17 percentage points less likely to be employed 6 months following diagnosis relative to women in the control group. Among women employed prior to diagnosis, 12% appeared to move out of the labor force altogether by retiring or becoming disabled [34]. The nonemployment effect of breast cancer appeared to be about twice as strong for African American women [34]. To put these findings in perspective, the American Cancer Society predicts 140000 cases of breast cancer in women under age 65 each year. If we estimate that 50% of these women are working, approximately 70000 women will experience labor market consequences each year attributable to breast cancer.

Research has found that men who are treated for prostate cancer have substantial complications that may interfere with their activities of daily living including their ability to work [35]. Research has shown that men with prostate cancer were less likely to be working 6 months following diagnosis relative to men without prostate cancer [36]. Cancer and its treatment interfered with some men's ability to perform physical and cognitive tasks once they returned to work. While early detection and treatment have positive implications for mortality, they may inflict morbidity—at least in the months immediately following treatment—that will interfere with patients' ability to work.

Few studies have measured absenteeism for those who remain employed while undergoing treatment and who return to their jobs after completing treatment. The Midlife Development in the United States Survey asked respondents questions about how many out of the past 30 days they were either totally unable to work or perform normal activities because of health problems (work loss days), or had to cut back on these activities because of health problems [37]. Although only 0.5% of the sample reported that they had cancer, cancer had the highest prevalence of any 30-day work impairment. Approximately 66% of those with cancer reported that the average number of days they were impaired was 16.4 [37]. These days were attributed to physical symptoms, primarily fatigue. It is interesting that employers often encourage their employees to use preventive health care services like cancer screening but are left in a quandary about how to manage an employee whose screening resulted in cancer detection. Likewise, physicians are left in a quandary when patients do not adhere to treatment regimens that interfere with their jobs.

## Scarcity of Internet Economic Information Available to Patients

Clearly, cancer patients and their caregivers already access and rely upon the Internet for information regarding treatment and advocacy. One study reports that 58% of cancer patients and their companions have access to the Internet from a home computer [38]. Patients and their companions routinely used information that described drugs, treatments, side effects, physicians, and hospitals.

Although there is a plethora of websites that provide cancer treatment information, few websites provide economic data. For example, Kelahan [39] reviewed 373 sites of organizations that sponsored clinical trial research, promoted patient advocacy, and oversaw clinical trials and found that less than 5% of them contained reimbursement information for medical expenses incurred under the auspices of the clinical trial. Without this critical economic information, patients cannot adequately evaluate their ability to participate in clinical trials.

A recent online article unfolded a story of rising costs of cancer drugs that extend life for only a few months beyond what can be achieved with standard therapies [40]. Drug costs alone can exceed US $250000 for a few months of treatment. Many patients may simply be unable to pay for these therapies—even if their out-of-pocket contributions are relatively low in comparison to the cost of care.

### Financial Assistance

Some websites offer assistance with regard to seeking financial resources. For example, the NCI website lists states that require health plans to cover patient care costs in clinical trials [41]. This same site offers a resource guide on clinical trials and insurance coverage that provides patients with procedures to follow for finding reimbursement for care provided under the auspices of clinical trials. It also lists organizations that provide financial assistance for cancer care. One commercial site, for example, provided a list of financial options for cancer patients on how they might receive funding for their health care [42]. Likewise, a number of charitable organizations have websites that direct patients on how to obtain supportive care products (eg, wigs, home health equipment). The American Cancer Society website offers a comprehensive description of how medical insurance, financial assistance, and cancer intersect [43]. This website not only lists organizations offering financial assistance, but it also makes suggestions to patients for becoming familiar with their insurance coverage, submitting insurance claims, and keeping records. This website attempts to explain government sponsored insurance programs and is to be applauded for explaining viatical or living benefits. It further offers extensive assistance for those who are uninsured. Finally, a nonprofit organization, Cancer Care, provides a list of programs offering financial assistance to patients with cancer [44].

### Treatment Costs

Taken together, the websites that broadly address cancer care and cost include information on clinical trials and insurance coverage, lists of organizations that provide financial assistance to patients with cancer, options for uninsured patients, and general guidance for seeking information regarding health insurance coverage. Absent from all of the websites reviewed is information that allows patients to estimate their costs prospectively so that they know and understand prior to seeking treatment the costs that they may incur. Although many patients are overwhelmed with their diagnosis, they require tools (eg, standardized worksheets, organizers) to help them plan for the expenses they may incur and to initiate discussions regarding cost with their providers before choosing a treatment path. Patients also require information on how to identify charges that are unrelated to their care and to alert their health care providers about inappropriate charges. Finally, patients need to be aware that they can negotiate with health care providers regarding payments and scheduling treatments so that the impact on work is lessened.

Brown et al lamented that acquisition of data to operationalize economic measures is far from complete [45]. This concern has been echoed throughout the literature (see [17] as an example). In a recent review of economic studies of cancer care, Fryback and Craig speculated that perhaps one day researchers will have standardized data collection tools and techniques to gather patient cost data. The means for collecting and documenting cost information, however, can be effectively and immediately placed in the hands of the patients and their families. The Internet is an ideal forum for exchanging information among patients regarding their care, for providing patient-centered worksheets for estimating costs, and for seeking assistance and resolution for charges. Without economic information, patients cannot make fully informed choices regarding their care.

### Work Loss

Research published in the scientific literature has linked cancer with substantial work loss. Yet, an Internet search of websites that address return-to-work issues for cancer survivors revealed a segmented approach to cancer treatment and returning to work. One site phrased its introduction to work issues as “When you're finally able to concentrate on something besides your cancer treatments, chances are you'll look forward to getting back to a more normal routine—this may mean going back to work” [46]. However, cancer treatment and employment are interdependent, rather than separate, occurrences. Many patients continue to work while undergoing the treatment. The website also provides considerable information on two policies particularly relevant to cancer patients—the Americans with Disabilities Act (ADA) and the Family Medical Leave Act (FMLA). The ADA requires employers to make “reasonable accommodation” for employees with a disability. The FMLA gives employees the right to take time off (up to 12 weeks of unpaid leave per year) due to their own illness, without the threat of losing their jobs.

Worthy of note is that the general tone of most websites describing the ADA and FMLA is litigious in nature. A legal perspective is partially relevant because many employed patients may be unaware that cancer is a condition covered by the ADA and their employers may inadvertently (or intentionally) violate the rights of these employees. However, a proactive, problem-solving approach to planning time away from work and to job restructuring could potentially be more constructive for patients than guidance on how to seek remediation after a violation has occurred. Patients need assistance with planning time away, negotiating with employers, and remaining in contact with employers and coworkers. Patients need to prepare for time away from work and should have reasonable expectations regarding their work performance while undergoing treatment. Patients who plan ahead may be more effective at negotiating with their employers and securing their jobs during treatment. Information on these topics is largely absent from the Internet—as well as other sources of patient information.

The many websites providing information on treatment, side effects, and methods for managing side effects make no mention of how treatment may interfere with patients' abilities to perform their jobs. Furthermore, many treatments have effects that may influence patients' job performance far into the future. The stimulus for work-related information may need to come from patients, advocacy groups, and government agencies. As cancer becomes a chronic condition, it is unrealistic and perhaps unwise to expect patients to quit their jobs altogether while undergoing treatment or to be unprepared for changes in job performance that extend beyond the active treatment period. Discussion about the integration of work and treatment along with strategies for lessening the burden of cancer and its treatment would be highly beneficial to cancer survivors and their families.

## Recommendations

This paper describes two important economic dimensions—medical costs and productivity costs—that are vital to patients diagnosed with cancer and to their families (Table 1). The Internet is a common means to convey information and assistance to those who are in need of guidance. While efforts have been made to translate scientific information regarding treatment, side effects, and outcomes to lay audiences, this effort has not expanded to the translation of economic data. Considerable opportunity exists to remedy the omission of economic information from credible websites, such as the ones sponsored by the NCI [41]. Information relevant to employed cancer patients, in particular, is sparse.

**Table 1:** Economic information needed by cancer patients

**Medical Costs**	**Productivity Costs**
Inpatient costs	Treatment side-effects specific to job performance
Outpatient costs	Expected absenteeism
Provider services	Protective laws and regulations
Supportive care	Strategies for negotiation with employer
Comparative treatment costs	Guidance for remaining employed
Insurance coverage	Guidance for understanding sick leave, vacation, and retirement benefits
Out-of-pocket costs	

Unfortunately, much of the information that would be helpful to patients does not yet exist, but it may become available in the future as more studies of the economic burden of cancer are sponsored. Nevertheless, intermediate steps can be taken toward providing information that may be very helpful to patients. First, websites containing clinical trial information should also contain cost and payment information. As part of this data, patients should be directed to explore payment options prior to enrolling in a trial or undertaking any treatment that may not be covered by their health insurance.

Second, websites and patient listservs that already provide a forum for patients to exchange information can be expanded to include the cost of care and help patients become more informed consumers (eg, [47]). These websites and online support groups offer an existing infrastructure for the collection, organization, and validation of cancer's economic costs.

Third, a website offering guidance to patients on how to organize their insurance information and charges for health care services by provider and date could be designed. This activity can help patients be more effective advocates for payment and readily address claims for service that have been denied by the health insurance. Charges and payment for health care services can be extraordinarily complicated and daunting under the best of circumstances. However, when faced with a potentially life-threatening disease requiring coordination of care across many providers, the task can be overwhelming for patients who are unprepared or less vigilant about ensuring that payment has been rendered for their health care.

Fourth, patients need information on how treatment may affect their ability to perform their jobs. Side effects of treatment are routinely described; however, the discussion of these side effects needs to be placed in the context of job performance. For example, statements about how fatigue may hinder some patients from performing theirs jobs, particularly if the job involves physical activities such as heavy lifting, walking, and standing for long periods of time, could be valuable to some patients who may not be aware that treatment may affect their job performance.

Fifth, patients require guidance on how much time away from work can be expected and how to proactively discuss absenteeism and job restructuring with their employer. Just as patients are encouraged to seek financial advice prior to initiating treatment, patients need to open communication with employers and coworkers about possible periods of absenteeism.

Finally, patients need to be encouraged to seek information on their sick leave, vacation, health insurance, and retirement benefits prior to initiating treatment. Without this information from their employer, patients may make decisions prematurely that can affect their future as well as their immediate economic well-being. In addition, through discussions with employers, coworkers, and other cancer survivors, patients may discover options for absenteeism and job restructuring that they had not previously considered. These recommendations are summarized in Table 2.

**Table 2:** Summary of recommendations

**Medical Costs**
Include cost information along with treatment information. Disclose the range of costs that patients may incur.
Provide a forum for patients to exchange information on medical costs and payment resolution. Capitalize on existing Internet infrastructure (eg, support groups, listservs, and chat rooms).
Guide patients on how to estimate costs and organize insurance information, provider charges, and payments.
**Productivity Costs**
Include information on how cancer treatment may affect job performance.
Offer guidance on expected absenteeism and how to plan and negotiate for time away from work.
Direct patients to explore health insurance, sick leave, vacation, and retirement benefits prior to initiating treatment.

Long-range plans for filling the void of economic information require further planning and execution. Some suggestions for how to proceed include the following: (1) formally assess patient needs for economic information; (2) sponsor studies to fill the void in information identified by patients; (3) sponsor the development of a specific site dedicated to economic information; (4) provide an online forum for patients to share their experiences in paying for care, resolving medical bills, and obtaining resources for payments and to share their work experiences, both positive and negative; (5) develop a range of strategies for negotiation with employers and planning time away from work; and (6) take measures to more fully understand and report the impact that cancer treatments have on patients' ability to work.

The President's Cancer Panel 2003 Annual Report identified several issues affecting cancer survivors across the life span [48]. Among these issues were the following: (1) cancer survivors and their families need better information about existing laws and regulations that may protect their employment, insurance, and assets; (2) education about cancer, cancer treatment, and survivorship needs is inadequate; and (3) existing insurance systems are an impediment to appropriate care for people with a cancer history. This final point is elaborated upon by stating that the link between employment and insurance disadvantages cancer survivors who risk losing both their employment and insurance during treatment. The Internet is a means by which to fill the gaps in information and to add the needed economic dimension to the discussion of cancer treatment and survivorship. The opportunity is substantial as the Internet can immediately put information in the hands of patients and their families—assisting them to become informed consumers and skilled negotiators—so that their economic viability can be preserved along with their lives.

## Abbreviations

ADA: Americans with Disabilities Act
FMLA: Family Medical Leave Act
NCI: National Cancer Institute
